# Sildenafil is a candidate drug for Alzheimer’s disease: Real‐world patient data observation and functional validation in patient iPSC‐derived neurons and the 5xFAD mouse model

**DOI:** 10.1002/alz.089662

**Published:** 2025-01-09

**Authors:** Yichen Li, Dhruv Gohel, Pengyue Zhang, Andrew A. Pieper, Jeffrey L. Cummings, Feixiong Cheng

**Affiliations:** ^1^ Cleveland Clinic, Cleveland, OH USA; ^2^ Indiana University, Bloomington, IN USA; ^3^ Case Western Reserve University, Cleveland, OH USA; ^4^ Chambers‐Grundy Center for Transformative Neuroscience, Department of Brain Health, School of Integrated Health Sciences, University of Nevada Las Vegas, Las Vegas, NV USA

## Abstract

**Background:**

Our previous study identified that Sildenafil (a phosphodiesterase type 5 [PDE5] inhibitor) is a candidate repurposable drug for Alzheimer’s Disease (AD) using *in silico* network medicine approach. However, the clinically meaningful size and mechanism‐of‐actions of sildenafil in potential prevention and treatment of AD remind unknown.

**Method:**

We conducted new patient data analyses using both the MarketScan® Medicare with Supplemental database (n = 7.23 million older [>65 years] subjects) and the OPTUM database (n = 11.52 million older subjects) to further test the real‐world evidence of sildenafil with AD incidence. We utilized the AD patient‐induced pluripotent stem cells (iPSC)‐derived neuron models (5 iPSC lines from both familial and sporadic AD patients) to investigate mechanism‐of‐action of sildenafil’s protective effects on AD. We also treated a transgenic AD mouse model (5xFAD) with 15 mg/kg sildenafil and conducted behavioral tests, immunofluorescence staining on mouse brain sections, and single‐cell RNA‐seq to evaluate the sildenafil’s efficacy and brain target engagement.

**Result:**

We found that sildenafil usage is significantly associated with reduced likelihood of AD across all four new drug cohorts (bumetanide, furosemide, spironolactone, and nifedipine). Specifically, sildenafil vs. spironolactone was associated with a 46% reduced prevalence of AD in MarketScan (HR = 54%, 95% CI 0.32‐0.66, p‐value = 3.33×10‐5) and a 30% reduced prevalence of AD in OPTUM (HR = 70%, 95% CI 0.49‐1.00, p‐value = 0.05). Sildenafil treatment reduces phosphorylated tau (pTau181 and pTau231), phosphorylated GSK‐3b and CDK5 in the AD patient‐iPSC‐derived neuron model. The 3 months sildenafil treatment significantly improved the cognition of 5xFAD transgenic mice on novel object recognition, Morris water maze and contextual fear conditioning test, supporting its therapeutic effects in AD from real‐world patient data and patient iPSC model findings. RNA‐seq analysis (bulk and single‐cell) revealed potential biomarkers and brain target engagement to be tested in future clinical trials.

**Conclusion:**

Those real‐world data, patient iPSC‐derived mechanic, and in vivo transgenic mouse model observations suggest that sildenafil offers a potential repurposable treatment for AD. However, future clinical trials are warranted to validate the causal relationship of sildenafil in potential prevention and treatment of AD.

